# Fractures of the Inferior Maxilla

**Published:** 1858-01

**Authors:** Frank H. Hamilton


// BUFFALO MEDICAL JOURNAL

AND

MONTHLY REVIEW.

VOL. 13. JANUARY, 1858. NO. 8.

### ORIGINAL COMMUNICATIONS.

### ART. I.—*Fractures of the Inferior Maxilla.* By Frank Hastings Hamilton, M. D.

(Continued from Page 397.)

*Treatment.* The few attempts which I have made to restore a completely dislocated tooth to its socket, or to retain it in place when very much loosened, have generally resulted in their removal at some later day, and especially where the fracture has been near the angle, and a molar has been disturbed. I believe it would be better practice always to remove the molars under these circumstances, unless they remain attached to the alveoli, and cannot be removed without bringing these away also: and this whether the loosened tooth is situated in the line of fracture or not. It is seldom that they can be made again to occupy their sockets perfectly, and where the tooth is in the line of the fracture the attempt to restore it to place will sometimes prevent the proper adjustment of the fragments. In cases, also, in which the teeth farther forward are completely dislodged at the seat of fracture, it is scarcely worth while to replace them.

As to those teeth whose loosened condition is due only to a splitting of the alveola, the same rule will not always apply. Sometimes, after a careful reädjustment, the fragments will reünite and the teeth remain firm.

If the alveola is broken only upon one side, through the line of the sockets, the teeth may not be always much disturbed, and the loss of the bony fragments may be of less consequence, nor have I generally succeeded in saving them; yet if they remain adherent to the soft parts, it is proper to make the attempt.

The expedients to which surgeons have resorted for the purpose of retaining in place the fragments, when the bone is broken through its body, may be arranged under the names of ligatures, splints, bandages and slings.

The ligature has been applied both to the teeth and to the bone itself. Thus, in an oblique fracture near the angle, where the fragments could not otherwise be prevented from falling inwards, Baudens passed a strong ligature, formed of thread, around the fragments and in immediate contact with them, tying the ligature over the teeth within the mouth. No accident followed, and on the twenty-third day, when he removed the ligature, the bone had united firmly and smoothly.*

In the case of the fracture of the Inferior Maxilla, reported by Dr. Buck, to the New York Pathological Society, and already referred to, the bone "was broken between the two incisor teeth of the left side; the part of the bone on the left of the fracture was driven in, and interlocked behind the end of the right portion, so as to be separated by a finger's breadth. Finding it impossible otherwise to reduce the fracture, Dr. B. dissected off the under lip, so as to expose the fracture. He found that the right anterior portion of the fractured bone terminated in an angular projection as far as on a line below the left angle of the mouth. The lip was then divided to the chin, and the soft parts holding the fragments together incised. A chisel was then insinuated behind the projecting angle of the bone, while it was being excised by the metacarpal saw. When the bone was restored to its natural position, it was found so apt to become displaced, that holes were drilled at the lower angle of the fracture, and adjustment maintained by wiring them together, the wire passing out through the lower angle of the wound. Sutures and adhesive straps, with a bandage, were employed to maintain the adjustment of the parts. So far the patient has done well, being supported by liquid nourishment introduced through a tube, passed

---

* Malgaigne, op. cit., p. 398.

through the space left by one of the incisors, which, on account of its looseness, was removed."*

With these two exceptions, so far as I am aware, the ligature has been employed as a means of retention only by fastening it upon the teeth, either upon those which are situated on the opposite sides of the fracture, or upon others a little more remote, or upon the corresponding teeth of the upper jaw, or upon the teeth on the opposite sides of the same jaw.

Ordinarily the ligature, composed of either fine gold, platinum or silver wire, or of firm silk or linen threads—(Celsus advised the use of horse hair)—has been applied to the two teeth on the opposite sides of the fracture, or if these have been not sufficiently firm, to the next teeth. This practise recommended first by Hippocrates, has received the occasional sanction of Ryff, Walner, Chelius, Lizars, Erichson, Miller, B. Cooper, Skey and others, but by Boyer, Gibson and Malgaigne it has been reprobated.

Dr. S. G. Ellis, of Gowanda, N. Y., as we have already seen, has, treated a fracture occurring through the symphysis, in an adult, by placing the mainspring of a watch within the dental arcade, and securing it in place with silver wire. The mouth was kept closed by bandages carried under the chin. The fragments united with only a slight vertical displacement.†

Dr. George Haywood, of Boston, surgeon to the Massachusetts General Hospital, says, "when the bone is not comminuted and there are teeth on each side of the fracture, the ends of the bone can be kept in exact apposition by passing a silver wire or strong thread around these teeth and tying it tightly. In several cases of fracture of the jaw, in which the bone was broken in one place only, I have in the course of the last few years, adopted this practice with entire success, and without the aid of any other means. It will be found very useful, also, as an auxillary, in more severe cases, in which it may be required to use splints and bandages, or to insert a piece of cork between the jaws, as recommended by Delpech. It requires some mechanical dexterity to apply the thread neatly; but in large cities we can avail ourselves of the skill of dentists for this purpose."‡

Guillaume de Salicet advises to secure, with a silk thread, at the same moment the teeth belonging to the two fragments, and the corresponding teeth of the upper jaw;§ while the dentist Lemaire, being applied to by

---

* New York Jour. of Med., &c., March, 1847, p. 211.

† Trans. Amer. Med. Assoc. My report on "Defor.," &c., vol. viii, p. 383, case 14.

‡ Boston Med. & Surg. Jour., vol. xix, p. 133, 1838.

§ Malgaigne, op. cit., p. 392.

Dupuytren to secure in place the ununited fragments of a broken jaw, fastened the two left canine teeth to each other by a wire of platinum, as had been already suggested by Guillaume de Salicet; to these he added two other modes of ligature which were altogether original. One wire, made fast to the last molar upon one side, traversed the mouth and was secured to one of the bicuspids upon the opposite side; the other was stretched from the first inferior bicuspid on the right to the first superior bicuspid on the left. A cure was accomplished at the end of two months, but one of the wires had nearly bisected the tongue; and as it had gradually become imbedded, the flesh had closed over it until it rested like a seton through the middle of the tongue.*

None of these various methods recommend themselves very satisfactorily to the practical surgeon; for besides that they are all of them, in a large majority of cases, wholly unnecessary, and in other cases, owing to the absence of the teeth, or to their loosened or decayed condition, or to the closeness with which they are set against each other, absolutely impossible, it must be seen, also, that they will generally prove feeble and inefficient. The wires act only upon the upper extremity of the line of fracture, leaving its lower portions liable to be disturbed by trivial causes; they tend gradually to loosen even the firm teeth which they embrace, and not unfrequently, after having been made fast with much labor, they soon become disarranged or break. They require therefore always the additional protection afforded by bandages. Alone they are insufficient, and if properly constructed bandages or slings are employed, they are not needed. Sometimes, moreover, they are actually mischievous, as when they loosen a sound tooth or press upon and inflame the gums. A. Berard passed a silver wire twice around the necks of two adjoining teeth on the opposite sides of a fracture. It retained the fragments perfectly in apposition during several days; but soon the gums swelled and became painful; the teeth loosened, and it was found necessary to remove the wire. Chassaignac sought to avoid these evils by placing the wire upon the middle of the crown, free from the gums, and by including four teeth instead of two. A waxed linen thread was made fast in this manner, in a case of simple fracture, on the seventh day. On the following morning the thread was found broken. He applied then a silk ligature in the same manner. On about the third day this also was disarranged; the ligatures were now discontinued until the eighteenth day, when he renewed the experiment with a piece of gold wire. Fourteen days

---

* Malgaigne, from Jour. Univer. des Sci. Med., tom. xix, p. 77.

after this the ligature remained firm, but the gums were red and bleeding. The patient not having again returned to Chaissaignac, the result is not known.*

As to the method suggested by Guillaume do Salicet it presents no advantages to compensate for its inconveniences; while that actually practiced by the dentist Lemaire, successful indeed, threatened to substitute a loss of the tongue for an ununited fracture of the jaw.

Splints have been employed in various ways. First, simple interdental splints, laid along the crowns of the teeth and only sufficiently grooved to be easily retained in place; Second, clasps, which are applied over the crowns and sides of the teeth, operating chiefly by their lateral pressure; Third, splints applied to the outer and inferior margin of the jaw; Fourth, interdental splints or clasps, combined with outside splints.

Interdental splints have been recommended by many surgeons from an early day, and they continue to be employed occasionally up to this moment.

Boyer advises the use of cork splints placed one on each side between the upper and lower jaws, in a few exceptional cases. Miller recommends the same in all cases, the "two wedges of cork sloping gently backwards, with their upper and under surfaces grooved for the reception of the upper and lower teeth." Fergusson also has usually adopted the same practice. Muys and Bertrandi employed ivory wedges.†

On the other hand, they are rejected entirely by Syme, Chelius, Skey, Erichsen, and Gibson.

The objections which have been stated to their use are,—that they are unsteady and become easily loosened and disarranged, that they occasionally press painfully upon the inside of the cheeks, that they accumulate about themselves an offensive sordes, and finally that they are unnecessary, since experience has proven, says Gibson, that "there is always sufficient space between the teeth to enable the patient to imbibe broth or any other thin fluid placed between the teeth."

It is not strictly true, however, that in all cases there will be found sufficient space between the teeth, when the mouth is closed, for the imbibition of nutrient fluids. I have myself seen exceptions, and in such a case the patient, if the mouth were closed in the usual way, would have to be fed through a tube conveyed along the nostrils into the stomach, as suggested by both Samuel and Bransby Cooper in certain bad compound fractures,

* Malgaigne, from Lond. Med. & Phys. Jour., Nov., 1822, p. 401.

† Lond. Med. Chi. Rev., vol. xx, p. 470.

or through an opening made by the extraction of one of the front teeth; neither of which methods ought to be preferred to the interdental splints; but then the separation of the front teeth for the purpose of receiving food, is by no means the only object to be gained by their use, nor indeed the principal object. Their great purpose is to act as splints whenever the absence of teeth either in the upper or lower jaw renders the two corresponding arcades unequal and irregular and prevents our making use of the upper jaw as a kind of internal splint for the lower jaw.

It is with a view to the accomplishment of this important end that they are often valuable, and ought sometimes to be considered as indispensable. I believe, also, that many of the inconveniences which have been found to attend the use of cork or wood, are obviated by the substitution of gutta percha in the manner which I have already recommended in my report to the American Medical Association, made in the year 1855. I have employed this method several times myself, and my suggestions have been followed by Stephen Smith, of the Bellevue Hospital, New York, who, after having used the gutta percha in four cases, affirms that nothing can surpass it in efficiency.

The mode of preparing gutta percha, and of adapting it between the teeth, is as follows: Dip a couple of pieces of the gum, of a proper size, into boiling water, and when they are sufficiently softened, mould them into wedge-shaped blocks, and, having wrapped each block with a piece of cotton cloth, carry them to their appropriate places between the back teeth; immediately press up each horizontal ramus of the jaw until the mouth is sufficiently closed, and the line of the inferior margin is straight; in this position retain the fragments a few minutes, until the gum has sufficiently hardened. Meantime, it will be practicable, generally, to introduce the fingers into the mouth, and to press the gutta percha laterally on each side towards the teeth, and thus to make its position more secure. When it is sufficiently hardened, remove the splints for the purpose of determining more precisely that they are properly shaped and fitted.

The superiority of this splint is now at once perceived. If properly made, it is smooth upon its surface, and not, therefore, so liable to irritate the mouth as wood or cork, and it is so moulded to the teeth that it will never become displaced.

The clasp, applied over the crowns and sides of the teeth is not intended to act as an interdental splint; but by its lateral pressure it is expected to hold the fragments in apposition upon nearly the same principle with the ligature.

Mütter, of Philadelphia, employs for this purpose, a plate of silver folded snugly over the tops and sides of two or more teeth adjacent to the fracture, which apparatus he calls a "clamp."*

Nicole, of Nuremburg, employed for the same purpose, a couple of steel plates fitted accurately along the anterior and posterior dental curvatures, secured in place by a steel clasp, the clasp being furnished with a thumb-screw in order the more effectually to accomplish the lateral pressure.

Malgaigne has extended the idea of Nicole, by substituting for the two steel plates, a single plate composed of flexible and ductile iron, which is fitted accurately to all the irregularities of the posterior dental arch. From the two extremities of this plate, and from two other intermediate points' four small steel shafts arise perpendicularly, cross the crowns of the teeth at right angles, and then fall down again perpendicularly upon the anterior dental arcade. Each steel shaft being furnished with a thumb-screw, the iron plate can now be made to bear against the teeth so as to form a posterior dental splint. The teeth are also protected in front against the direct action of the thumb-screw by the interposition of a leaden plate.

I am not aware that either of these modes have ever been practically tested; and I confess that I can see many disadvantages and inconveniences which would be likely to arise from their use. With the exception of Mütter's "clamp," they are all complex and must be liable to disarrangement; while thumb-screws in the mouth cannot but inflict serious injury by their pressure and friction against the mucous membrane.

Gutta percha employed in the manner which I have recommended, is capable of giving no inconsiderable degree of lateral support to the teeth, and I suspect quite as much as the comfort or interest of the patient will permit, and without many of the inconveniences of the other modes, while it possesses the additional advantage of serving also where this is needed, as an efficient interdental splint.

External splints, applied along the base or outside of the jaw, were first recommended by Paré, who used for this purpose, leather; and they have been employed in some form, occasionally, by most surgeons. Generally they have been composed of flexible materials, such as wetted pasteboard, first recommended by Hesiter, felt, linen saturated with the whites of eggs, paste, dextrine or starch; plaster of Paris has also been used: and they have been retained in place by either bandages or the sling. I have myself used

* Trans. Am. Med. Assoc., vol. viii, p. 391. My report on Deformities, &c.

for this purpose, gutta percha, but I shall speak of it as one form of the sling dressing.

Undoubtedly useful, and even necessary in some cases, especially where there exists a great tendency to a vertical displacement, they will be found, also, in many cases, to render no essential service, and may properly enough be dispensed with.

Whatever objections hold to the use of metallic clasps, must hold equally to the use of those forms of apparatus in which it is attempted to secure the fragments by means of a combination of these clasps with outside splints, and in which it is proposed to dispense with all bandages or slings, the mouth being permitted to open and close freely during the whole treatment. They are liable, moreover, to additional objections which will be readily suggested by an explanation of their mode of construction.

Chopart and Desault originated this idea as early as 1780, for fractures occurring upon both sides; in which cases they advised "bandages composed of crotchets of iron or of steel, placed over the teeth, upon the alveolar margin, covered with cork or with plates of lead, and fastened by thumbscrews to a plate of sheet iron, or to some other material under the jaw."

The apparatus invented by Rutenick, a German surgeon, in 1799, and improved by Kluge, is thus described by Dr. Chester: "It consists, 1st, of small silver grooves, varying in size according as they are to be placed on the incisors or molars, and long enough to extend over the crowns of four teeth; 2d, of a small piece of board, adapted to the lower surface of the jaw, and in shape resembling a horse shoe, having at its two horns, two holes on each side; 3d, of steel hooks of various sizes, each having at one extremity an arch for the reception of the lower lip, and another smaller for securing it over the silver channels on the teeth, and at the other a screw to pass through the horse shoe splint and to be secured to it by a nut and a horizontal branch at its lower surface; 4th, of a cap or silk nightcap to remain on the head; and 5th, of a compress corresponding in shape and size with the splint. The net or cap having been placed on the head and the two straps fastened to it on each side, one immediately in front of the ear and the other about three inches farther back, which are to retain the splint in its position by passing through the two holes in each horn; a silver channel is placed on the four teeth nearest to the fracture, on this the small arch of the hook is placed, and the screw end having been passed through a hole in the splint is screwed firmly to it by the nut, after a compress has been placed between the splint and the integuments below the jaw.

"If there is a double fracture, two channels and two hooks, must, of course, be used."*

Bush invented a similar apparatus in 1822,† and Houzelot in 1826; since which the apparatus has been variously modified by Jousset, Lonsdale, Malgaigne and perhaps others.

Lonsdale says he has employed his instrument in numerous cases and with complete success.‡ Rutenick sncceeded with his apparatus in a case where the displacement persisted in spite of all other means.§ Jousset was also successful in two cases.‖

But others have not been equally fortunate; or if they have succeeded in holding the fragments in apposition, and in securing a bony union, other serious accidents have followed.

In the first case mentioned by Houzelot, the instrument was kept on thirteen days, after which an attack of epilepsy deranged every thing, and the patient was transferred to Bicetre. The second patient complained immediately of an intense pain under the chin and a profuse salivation followed. These symptoms were subdued by the sixth day, but, for some reason, the apparatus was finally removed on the tenth day. The fragments hereafter showed no tendency to derangement. Seven days after its removal an abscess, which had formed under the chin, was opened. In the third case the apparel was left in place thirty days, and an abscess formed also under the chin. Neucourt applied it in a double fracture where the central fragment was much displaced. The apposition was well preserved, but he was obliged to remove it on the seventeenth day on account of a phlegmon which was forming under the chin. The patient to whom Bush applied his apparatus, would wear it but a few days. Malgaigne had the same experience with Bush's apparatus.

In addition to the pain and inflammation, followed by submaxillary abscesses, which have been such frequent results of its use, Malgaigne has noticed that it is exceedingly inclined to slide forwards and become displaced.

In short, notwithstanding the unqualified testimony of Lonsdale in favor of this method of treatment, especially in fractures at the symphysis, and in fractures through any portion of the shaft anterior to the masseter muscle, it

---

* London Med. Chi. Rev., vol. xx, p. 471, from Monthly Archives of the Medical Sciences, 1834.

† Malgaigne, op. cit., p. 395.

‡ Lonsdale. Practical Treatise on Fractures; London, 1838, p. 234.

§ Malgaigne, op. cit., p. 396.

‖ Ibid, p. 396.

is, in my judgment, sufficiently plain that it is applicable to only a very limited number of cases, and I am not certain but that it would be better to reject it altogether; and I should scarcely have thought it worth while to notice these modes of treatment at all were it not for the respectability of the gentlemen who have given them their countenance, and perhaps to show how fruitful and exhaustless in resources is the genius of our profession.

The treatment of fractures of the inferior maxilla by a single headed bandage or roller, numbers among its distinguished advocates the names of Gibson, Barton and H. H. Smith; indeed I think the practice is at the present time, peculiar to a few American surgeons. Gibson gives the following directions for applying his roller: "A cotton or linen compress, of moderate thickness, reaching from the angle of the jaw nearly to the chin, is placed beneath and held by an assistant, while the surgeon takes a roller, four or five yards long, an inch and a-half wide, and passes it by several successive turns under the jaw, up along the sides of the face and over the head; now changing the course of the bandage, he causes it to pass off at a right angle from the perpendicular cast, and to encircle the temple, occiput and forehead, horizontally, by several turns; finally, to render the whole more secure, several additional horizontal turns are made around the back of the neck, under the ear, along the base of the jaw, over the point of the chin. To prevent the roller from slipping or changing its position, a short piece may be secured by a pin to the horizontal turn that encircles the forehead, and passed backwards along the center of the head as far as the neck, where it must be tacked to the lower horizontal turn — taking care to fix one or more pins at every point at which the roller has crossed."

Barton employs, also, a compress, and a roller five yards long; the application of which is thus described by Sargent: "Place the initial extremity of the roller upon the occiput, just below its protuberance, and conduct the cylinder obliquely over the center of the left parietal bone to the top of the head; thence decend across the right temple and the zygomatic arch, and pass beneath the chin to the left side of the face; mount over the left zygoma and temple to the summit of the cranium, and regain the starting point at the occiput by traversing obliquely the right parietal bone; next wind around the base of the lower jaw on the left side to the chin, and thence return to the occiput along the right side of the maxilla; repeat the same course, step by step, until the roller is spent, and then confine its terminal end."

These bandages possess the advantages of being easily obtained, of symplicity and facility of application, and in general, we may add, of complete adaptation to the ends proposed. The only objections to their use which I have ever noticed, are occasional disarrangements, and the tendency, as in all other continuous rollers, to draw the fragments to one side or the other, according as the successive turns of the bandage are carried to the right or left. There is one other objection, having reference to the occasional inadequacy of this dressing to prevent an overlapping of the fragments, to which also the sling as usually constructed, is equally obnoxious, and of which I shall speak presently.

Finally, it is to the sling, in some of its various forms that surgeons have generally given the preference. The sling is known, also, by the name of the four headed or the four tailed roller or bandage.

B. Bell, Boyer, Skey, S. Cooper, B. Cooper, Syme, Fergusson, Mayor, Lizars and Chelius, employ the sling usually; and the favorite mode is to use for this purpose a piece of muslin cloth about one yard long and four inches wide, torn down from its two extremities to within about three or four inches of the center. Others have used leather, gutta percha, adhesive straps, gum elastic, etc.

Where the muslin is used, it is quite customary to lay against the skin a piece of pasteboard, wetted and moulded to the chin, or simply a soft compress; and some choose to open the center of the bandage sufficient to receive the chin. The middle of this bandage being laid upon the chin, the two ends corresponding to the upper margin of the roller are now carried across the front of the chin, behind the nape of the neck, and made fast; while the two lower heads are brought directly upwards from under the sides of the chin, along the sides of the face, in front of the ears, and made fast upon the top of the head. The dressing is completed by a short counter-band extending across the top of the head from one bandage to the other; or the several bands may be made fast to a night cap, in which case the counter-band will be unnecessary.

It only remains for me to describe my own method of dressing these fractures with the sling.

Having frequently noticed the tendency of the sling, as ordinarily constructed, and of Gibson's roller, to carry the anterior fragment backwards, especially in double fractures, where the body of the bone is broken upon both sides, I devised, some years since, an apparatus intended to obviate this objection, and which I have used now several times with complete success.

It is composed of a firm leather strap, called maxillary, which, passing perpendicularly upwards from under the chin, is made to buckle upon the top of the head, at a point near the situation of the anterior fontanelle. This strap is supported by two counter-straps, called, respectively, occipital and frontal, made of strong linen webbing. One of these, the occipital, is attached to the posterior margin of the maxillary strap about half an inch above the ear, and being carried around behind and *under* the occiput, it is finally buckled to the maxillary strap upon the opposite side, and at a point exactly corresponding to its origin. The frontal stay simply antagonizes the occipital; and having its origin and termination at the anterior margins of the maxillary strap, it is buckled horizontally across the forehead, and just above the eyebrows.

The maxillary strap is narrow under the chin to avoid pressure upon the front of the neck, but immediately becomes wider so as to cover the sides of the inferior maxilla and face, after which it gradually diminishes to accommodate the buckle upon the top of the head. The anterior margin of this band, at the point corresponding to the symphysis menti, and for about two inches on each side, is supplied with thread holes, for the purpose of attaching a piece of linen which, when the apparatus is in place, shall cross in front of the chin, and prevent the maxillary strap from sliding backwards against the front of the neck.

The advantage of this dressing over any which I have yet seen, consists in its capability to lift the anterior fragment almost vertically, and at the same time it is in no danger of falling forwards and downwards upon the forehead. If, as in the case of most other dressings, the occipital stay had its attachment opposite to the chin, its effect would be to draw the central fragment backwards. By using a firm piece of leather as a maxillary band, and attaching the occipital stay above the ears, this difficulty is completely avoided,

Having removed such teeth as are much loosened at the point of fracture, and replaced those which are loosened at other points, unless it be far back in the mouth, and adjusted the fragments accurately, the lower jaw is to be closed completely upon the upper, and the apparatus snugly applied. It is not necessary, in most cases, to buckle the straps with great firmness, since experience has shown that a sufficient degree of immobility is obtained when the apparatus is only moderately tight. In this matter I am sustained also by the opinion of Mr. Fergusson.

If the integuments are bruised and tender, a compress made of two or more thickness of patent lint should be placed underneath the chin, between it and the leather.

If the inability to introduce nourishment between the teeth when the mouth is closed, or the irregularity of the dental arcade renders the use of interdental splints necessary, gutta percha, as I have already explained, ought to be preferred to any other material.

The patient must be forbidden to talk, or laugh, and when he lies down his head should rest upon its back, for whatever mode of dressing is employed, and however carefully it is applied, it will be found that a slight motion and displacement will occur whenever the weight of the head rests upon the side of the face.

Occasionally, indeed as often as every two or three days, the apparatus may be loosened or removed, only taking care generally not to disturb the interdental splints, when they are used, and to support the jaw with the hand, during its removal, and the face may be sponged off with warm water and castile soap. It should not be left off entirely, however, in less than three or four weeks, where the fracture is most simple, nor ought the patient to be allowed to eat meat in less than four or five weeks.

To cleanse the mouth and prevent offensive accumulations, it should be washed several times a day with a solution of tincture of myrrh, prepared by adding one drachm to about four ounces of water.

The same apparatus, and without any essential modification, is applicable to fractures of the symphysis and of the angle of the inferior maxilla, as well as to fractures of the body of the bone.

Instead of the leather, I have in a few instances, especially of compound fractures, where it became necessary to allow the pus to discharge externally, used a sling or a splint composed of gutta percha, suspended by bands carried over the top of the head. The piece from which this splint is made should be two or three lines in thickness, covered with cloth, and padded under the chin. It will be found convenient to cover it with cloth before immersing it in the hot water. The water should be nearly at a boiling temperature, so that the splint may become perfectly pliable; and it should be laid upon the face and allowed to mould itself while the patient lies upon his back.

Having thus fitted it accurately to the face, it may be removed and openings made at points corresponding with the wounds upon the skin, before it is reäpplied.

In fractures of either condyle, unaccompanied with displacement, the simple leather or muslin sling will sometimes accomplish a perfect and speedy cure,as the two cases reported by Desault will sufficiently demonstrate. But if the fragments have become separated, the replacement is difficult, and the retention uncertain.

Ribes was the first to suggest and to practice the only rational method of reduction in these cases. Having seen two examples which had resulted in deformity under the usual treatment, which consisted in simply pressing forward the angle of the jaw, it occurred to him that while the upper or condyloidean fragment was not acted upon at the same moment by pressure from the opposite direction, a reduction must be imposible. The case of a canonnier whose jaw was broken through the neck of the condyle on the right side, and through its body on the left, afforded him an opportunity to determine the practicability of a method of which he had as yet only conceived the idea. Malgaigne thus describes his procedure: With the left hand seize the anterior portion of the jaw, for the purpose of drawing it horizontally forwards, while you carry the index finger of the right hand to the lateral and superior part of the pharynx. You will meet at first the projection formed by the styloid process, but moving your finger forwards you will find soon the posterior border of the ramus of the jaw; and following this border from below upwards, you will arrive at the inner side of the condyle which you will push outwards in such a manner as to engage it upon the other fragment. This manœuvre cannot be made without causing nausea as the finger always does when carried into the posterior part of the pharynx; but this is a slight inconvenience. The reduction obtained, bear the jaw upwards and backwards in order to press and fix the condyle between it and the glenoid cavity, then fasten it in place with the sling." The fragments were thus easily brought into apposition in the case reported by Ribes, and the patient was cured without any deformity.

In addition to these means, the angle of the jaw ought to be pressed permanently forwards by means of a compress placed between it and the mastoid process, and held in place by a suitable bandage.

If the coronoid process be alone broken, it is sufficient to close the mouth with any form of sling or bandage which may be most convenient.

